# What is next generation sequencing?

**DOI:** 10.1136/archdischild-2013-304340

**Published:** 2013-08-28

**Authors:** Sam Behjati, Patrick S Tarpey

**Affiliations:** 1 Cancer Genome Project, Wellcome Trust Sanger Institute, Wellcome Trust Genome Campus, Hinxton, Cambridgeshire, UK; 2 Department of Paediatrics, University of Cambridge, Cambridge, UK

**Keywords:** Technology, Basic Science

## Abstract

Next generation sequencing (NGS), massively parallel or deep sequencing are related terms that describe a DNA sequencing technology which has revolutionised genomic research. Using NGS an entire human genome can be sequenced within a single day. In contrast, the previous Sanger sequencing technology, used to decipher the human genome, required over a decade to deliver the final draft. Although in genome research NGS has mostly superseded conventional Sanger sequencing, it has not yet translated into routine clinical practice. The aim of this article is to review the potential applications of NGS in paediatrics.

## Introduction

**Figure 1:**
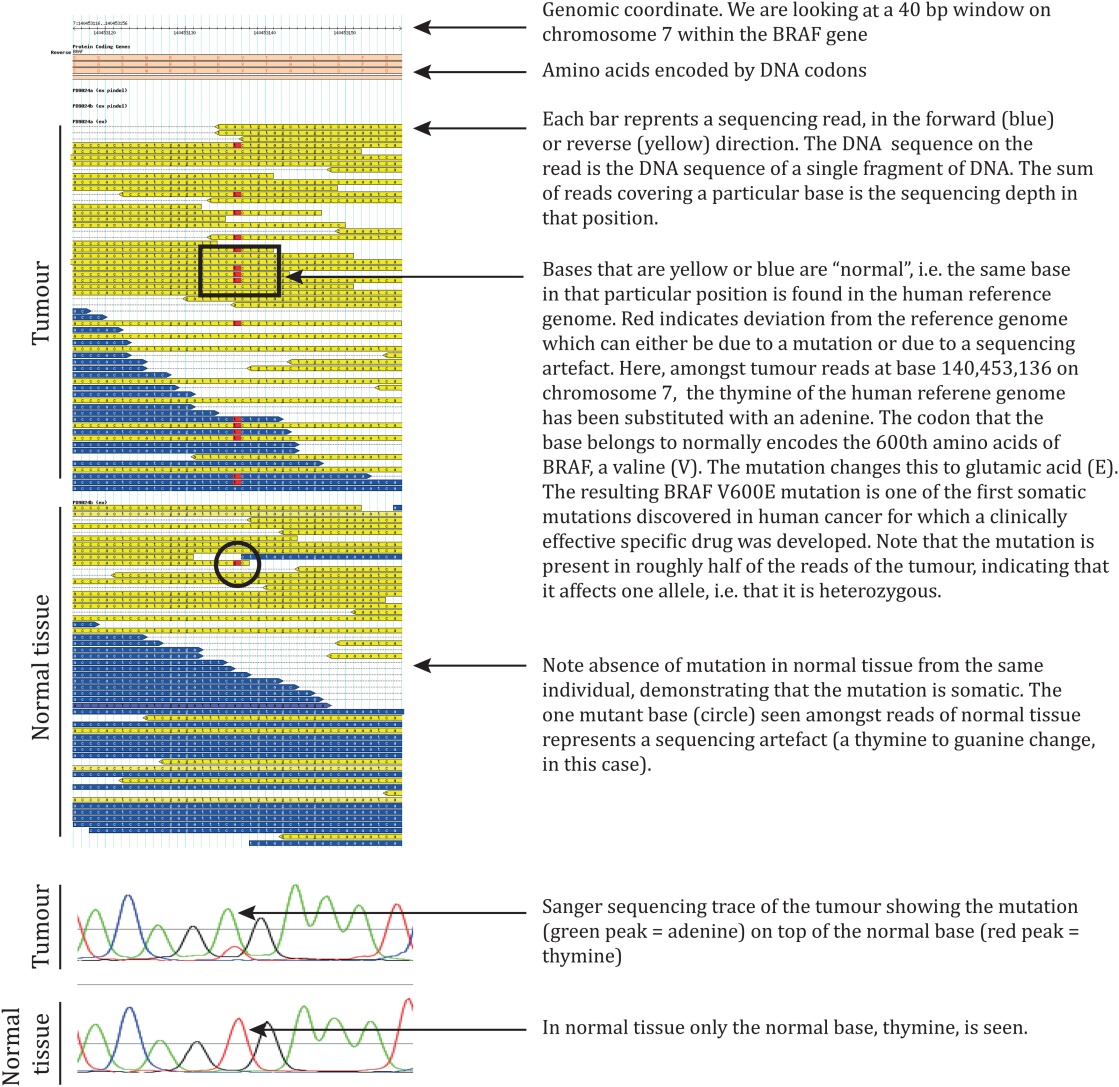
Example of next generation sequencing (NGS) raw data-BRAF V600E mutation in melanoma. The mutation was found by our group in 2002 as part of several year-long efforts to define somatic mutations in human cancer using Sanger sequencing, prior to the advent of NGS.

There are a number of different NGS platforms using different sequencing technologies, a detailed discussion of which is beyond the scope of this article. However, all NGS platforms perform sequencing of millions of small fragments of DNA in parallel. Bioinformatics analyses are used to piece together these fragments by mapping the individual reads to the human reference genome. Each of the three billion bases in the human genome is sequenced multiple times, providing high depth to deliver accurate data and an insight into unexpected DNA variation (figure 1). NGS can be used to sequence entire genomes or constrained to specific areas of interest, including all 22 000 coding genes (a whole exome) or small numbers of individual genes.

## Potential uses of NGS in clinical practice

### Clinical genetics

There are numerous opportunities to use NGS in clinical practice to improve patient care, including:

#### NGS captures a broader spectrum of mutations than Sanger sequencing

The spectrum of DNA variation in a human genome comprises small base changes (substitutions), insertions and deletions of DNA, large genomic deletions of exons or whole genes and rearrangements such as inversions and translocations. Traditional Sanger sequencing is restricted to the discovery of substitutions and small insertions and deletions. For the remaining mutations dedicated assays are frequently performed, such as fluorescence in situ hybridisation (FISH) for conventional karyotyping, or comparative genomic hybridisation (CGH) microarrays to detect submicroscopic chromosomal copy number changes such as microdeletions. However, these data can also be derived from NGS sequencing data directly, obviating the need for dedicated assays while harvesting the full spectrum of genomic variation in a single experiment. The only limitations reside in regions which sequence poorly or map erroneously due to extreme guanine/cytosine (GC) content or repeat architecture, for example, the repeat expansions underlying Fragile X syndrome, or Huntington's disease.

#### Genomes can be interrogated without bias

Capillary sequencing depends on preknowledge of the gene or locus under investigation. However, NGS is completely unselective and used to interrogate full genomes or exomes to discover entirely novel mutations and disease causing genes. In paediatrics, this could be exploited to unravel the genetic basis of unexplained syndromes. For example, a nationwide project, Deciphering Developmental Disorders,^1^ running at the Wellcome Trust Sanger Institute in collaboration with NHS clinical genetics services aims to unravel the genetic basis of unexplained developmental delay by sequencing affected children and their parents to uncover deleterious de novo variants. Allying these molecular data with detailed clinical phenotypic information has been successful in identifying novel genes mutated in affected children with similar clinical features.

#### The increased sensitivity of NGS allows detection of mosaic mutations

Mosaic mutations are acquired as a postfertilisation event and consequently they present at variable frequency within the cells and tissues of an individual. Capillary sequencing may miss these variants as they frequently present with a subtlety which falls below the sensitivity of the technology. NGS sequencing provides a far more sensitive read-out and can therefore be used to identify variants which reside in just a few per cent of the cells, including mosaic variation. In addition, the sensitivity of NGS sequencing can be increased further, simply by increasing sequencing depth. This has seen NGS employed for very sensitive investigations such as interrogating foetal DNA from maternal blood^2^ or tracking the levels of tumour cells from the circulation of cancer patients.^3^

## Microbiology

The main utility of NGS in microbiology is to replace conventional characterisation of pathogens by morphology, staining properties and metabolic criteria with a genomic definition of pathogens. The genomes of pathogens define what they are, may harbour information about drug sensitivity and inform the relationship of different pathogens with each other which can be used to trace sources of infection outbreaks. The last recently received media attention, when NGS was used to reveal and trace an outbreak of methicillin-resistant Staphylococcus aureus (MRSA) on a neonatal intensive care unit in the UK.^4^ What was most remarkable was that routine microbiological surveillance did not show that the cases of MRSA that occurred over several months were related. NGS of the pathogens, however, allowed precise characterisation of the MRSA isolates and revealed a protracted outbreak of MRSA which could be traced to a single member of staff.

## Oncology

The fundamental premise of cancer genomics is that cancer is caused by somatically acquired mutations, and consequently it is a disease of the genome. Although capillary-based cancer sequencing has been ongoing for over a decade, these investigations were limited to relatively few samples and small numbers of candidate genes. With the advent of NGS, cancer genomes can now be systemically studied in their entirety, an endeavour ongoing via several large scale cancer genome projects around the world, including a dedicated paediatric cancer genome project.^5^ For the child suffering from cancer this may provide many benefits including a more precise diagnosis and classification of the disease, more accurate prognosis, and potentially the identification of ‘drug-able’ causal mutations. Individual cancer sequencing may, therefore, provide the basis of personalised cancer management. Currently pilot projects are underway using NGS of cancer genomes in clinical practice, mainly aiming to identify mutations in tumours that can be targeted by mutation-specific drugs.

## Limitations

The main disadvantage of NGS in the clinical setting is putting in place the required infrastructure, such as computer capacity and storage, and also the personnel expertise required to comprehensively analyse and interpret the subsequent data. In addition, the volume of data needs to be managed skilfully to extract the clinically important information in a clear and robust interface. The actual sequencing cost of NGS is negligible. For example, a state of the art NGS platform can generate approximately 150 000 000 reads for around £1000 whereas a single Sanger read typically costs less than £1. However, to make NGS cost effective one would have to run large batches of samples which may require supra-regional centralisation. Following the initial capital investment, the capacity of a NGS facility can provide a service on national scale likely offering economic benefits in addition to improvements in patient care.

**Clinical bottom line**

- NGS has huge potential but is presently used primarily for research.
- NGS will allow paediatricians to take genetic information to the bedside.
